# Bimodal antibody-titer decline following BNT162b2 mRNA anti-SARS-CoV-2 vaccination in healthcare workers of the INT – IRCCS “Fondazione Pascale” Cancer Center (Naples, Italy)

**DOI:** 10.1186/s13027-022-00451-1

**Published:** 2022-07-28

**Authors:** Maria Antonietta Isgrò, Giusy Trillò, Luigi Russo, Anna Lucia Tornesello, Luigi Buonaguro, Maria Lina Tornesello, Leonardo Miscio, Nicola Normanno, Attilio Antonio Montano Bianchi, Franco Maria Buonaguro, Ernesta Cavalcanti, the anti-COVID-19 INT Task Force, Domenica Rea, Lucia Di Capua, Francesco Labonia, Serena Meola, Annamaria Piscopo, Sergio Arpino, Carmine Di Napoli, Gerardo Esposito, Vincenzo Pane, Valentina Delle Donne, Noemi Starita, Andrea Cerasuolo, Mariella Tagliamonte, Egidio Celentano, Anna Crispo, Concetta Montagnese, Giuseppe Porciello, Emanuela Rotondo, Roberto Simioli, Maria Grimaldi, Flavia Nocerino, Paola Murino, Stefania D’Auria, Rocco Saviano

**Affiliations:** 1 Istituto Nazionale Tumori – IRCCS, Fondazione Pascale, Naples, Italy; 2 Division of Laboratory Medicine, Istituto Nazionale Tumori – IRCCS, Fondazione Pascale, Naples, Italy

**Keywords:** Bimodal titer, BNT162b2 (Pfizer-BioNTech), Antibody avidity, Immunoprotective titer

## Abstract

**Background:**

Both SARS-CoV-2 mRNA-based vaccines [BNT162b2 (Pfizer-BioNTech) and mRNA-1273 (Moderna)] have shown high efficacy, with very modest side effects in limiting transmission of SARS-CoV-2 and in preventing the severe COVID-19 disease, characterized by a worrying high occupation of intensive care units (ICU), high frequency of intubation and ultimately high mortality rate. At the INT, in Naples, only the BNT162b2/Pfizer vaccine has been administered to cancer patients and healthcare professionals aged 16 and over. In the present study, the antibody response levels and their decline were monitored in an interval of 6–9 months after vaccine administration in the two different cohorts of workers of the INT – IRCCS "Fondazione Pascale" Cancer Center (Naples, Italy): the group of individuals previously infected with SARS-CoV-2 and vaccinated with a single dose; and that of individuals negative for previous exposure to SARS-CoV-2 vaccinated with two doses 21 days apart.

**Methods:**

Specific anti-RBD (receptor-binding domain) titers against trimeric spike glycoprotein (S) of SARS-CoV-2 by Roche Elecsys Anti-SARS-CoV-2 S ECLIA immunoassay were determined in serum samples of 27 healthcare workers with a previously documented history of SARS-CoV-2 infection and 123 healthcare workers without, during antibody titers’ monitoring. Moreover, geometric mean titers (GMT) and relative fold changes (FC) were calculated.

**Results:**

Bimodal titer decline was observed in both previously infected and uninfected SARS-CoV-2 subjects. A first rapid decline was followed by a progressive slow decline in the 6/9 month-period before the further vaccine boost. The trend was explained by 2 different mathematical models, exponential and power function, the latter revealing as predictive of antibody titer decline either in infected or in not previously infected ones. The value of the prolonged lower vaccine titer was about 1 log below in the 6/9-month interval after the single dose for previously infected individuals with SARS-CoV-2 and the two doses for those not previously infected. The titer change, after the boost dose administration, on the other hand, was ≥ 1.5 FC higher than the titers at the 6/9-month time-points in both cohorts. A similar quantitative immune titer was observed in both cohorts 8 days after the last boost dose. The subsequent immunoresponse trend remains to be verified.

**Discussion:**

The results show that a very rapid first decline, from the highest antibody peak, was followed by a very slow decline which ensured immune protection lasting more than 6 months. The apparent absence of adverse effects of the rapid decline on the vaccine's immune protective role has been related to a large majority of low avidity antibodies induced by current vaccines. High avidity antibodies with prolonged anti-transmission efficacy show a longer half-life and are lost over a longer interval period. The cellular immunity, capable of preventing severe clinical diseases, lasts much longer. The unbalanced dual activity (cellular *vs* humoral) while effective in limiting ICU pressure and overall mortality, does not protect against transmission of SARS-CoV-2, resulting in high circulation of the virus among unvaccinated subjects, including the younger population, and the continuous production of variants characterized by changes in transmissibility and pathogenicity. The high mutation rate, peculiar to the RNA virus, can however lead to a dual opposite results: selection of defective and less efficient viruses up to extinction; risk of more efficiently transmitted variants as the current omicron pandemic.

**Conclusions:**

In conclusion the current bimodal antibody-titer decline, following BNT162b2 mRNA anti-SARS-CoV-2 vaccination, needs a further extended analysis to verify the protective borderline levels of immunity and the optimal administration schedule of vaccine boosters. Our current results can contribute to such goal, besides a direct comparison of other FDA-approved and candidate vaccines.

## Background

Severe Acute Respiratory Syndrome Coronavirus-2 (SARS-CoV-2), a positive-sense single-stranded RNA virus belonging to the Coronaviridae family, causes coronavirus disease 2019 (COVID-19), a respiratory syndrome evolving frequently to severe pneumonia, respiratory and multi-visceral failure and causing death in some patients with comorbidity [1].

Preventive protection against many infectious diseases is mediated by a functional, persistent antibody response, which is therefore a critical immune correlate for many licensed human vaccines. The durability of vaccine-acquired antibody responses varies greatly among antigens [2]. Antibodies induced by viral infections, or by vaccination with live-attenuated viruses, can persist for decades. However, most vaccines based on protein antigens require repeated immunizations to generate immunological memory, and to maintain antibody responses above protective levels [3]. The level of antigen–antibody binding avidity, a qualitative response index, can also correlate with protection. Nevertheless, low-avidity antibodies have been associated with antibody-mediated disease enhancement following pandemic influenza vaccinations [4, 5].

Moreover, inadequate levels of avidity maturation (the latter defined as the increase of avidity over time) can heighten susceptibility to viral infection [6]. Thus, both quantitative and qualitative yardsticks can determine vaccine efficacy. Vaccine adjuvants are linked to both of these aspects of the antibody response. By enhancing innate immunity, they promote activation of naïve B cells and CD4 + T cells [7].

The immune system represents an important component against the viral infection by the titer and avidity of neutralizing antibodies production. The trimeric spike glycoprotein (S) of SARS-CoV-2 is a key target for virus neutralizing antibodies and the prime candidate for vaccine development [8]. The protein S binds its cellular receptor on the host cells, human angiotensin converting enzyme 2 (ACE2), through a receptor-binding domain (RBD) [9].

Both SARS-CoV-2 mRNA-based vaccines containing the messenger RNA that encodes the SARS-CoV-2 S in small lipid particles [BNT162b2 (Pfizer-BioNTech) and mRNA-1273 (Moderna)] have shown high efficacy, with very modest side effects in limiting transmission of SARS-CoV-2 and in preventing the severe COVID-19 disease, characterized by a worrying high occupation of intensive care units (ICU), high frequency of intubation and ultimately high mortality rate.

On 22nd December 2020, the Italian regulatory agency for drugs AIFA (Agenzia Italiana del Farmaco) authorized in Italy the use of BNT162b2/Pfizer vaccine, in 2 doses with an interval of 21 days between the doses [10]. Recent studies have found that subjects infected with COVID-19 develop higher antibody titers after vaccination [11] and present protective immunity for at least 6 months [12], but the impact of previous exposure to SARS-CoV-2 on immune response elicited by the vaccines needs to be further verified in larger trial studies. Several studies have shown that the immune response to the vaccine after the first dose is substantially more pronounced in individuals with pre-existing immunity and it is similar to the immune response developed after the second dose in individuals not previously infected [11, 13–15]. However, few data are available on the accurate monitoring of the titers’ decline, response to further boosters, optimal vaccine dosage and role of different adjuvants on vaccine efficacy.

In the present study, the antibody response levels and their decline were monitored in an interval of 6/9 months after vaccine administration in the two different cohorts of workers of the INT – IRCCS "Fondazione Pascale" Cancer Center (Naples, Italy), established since 2020 [16]: the group of individuals previously infected with SARS-CoV-2 and vaccinated with a single dose; and that of individuals negative for previous exposure to SARS-CoV-2 vaccinated with two doses 21 days apart.

## Materials and methods

### Sample size

According to our national recommendations, the internal health surveillance program for healthcare workers implemented the following BNT162b2/Pfizer vaccination schedule: 1 dose was administered to subjects previously infected to SARS-CoV-2, and 2 doses (with an interval of 21 days) to subjects not infected. Most of healthcare providers underwent also boost dose. The program contemplated the evaluation of antibody responses by determining anti-RBD titers at three times: the day before vaccination (baseline anti-S antibody titer), 20 days after the first dose and 8 days after the second dose for not previously infected workers and 8 days after the unique dose for previously infected healthcare providers. Further data were collected by determining antibody titers 1 month after the preliminary completion of vaccination cycle and 8 days after the boost dose administration for both cohorts. Time points were defined as follows:

- For not infected subjects:
  - T0 = baseline pre-vaccination.
  - T1 = 20 days after the first dose.
  - T2 = 8 days after the second dose, distinguishing:
    - T2 I = 8 days after the second dose.
    - T2 II = 1 month after the second dose.
  - T3 = 3 months after the second dose.
  - T6 = 6 months after the second dose.
  - T9 = 9 months after the second dose.
  - T boost = 8 days after the administration of a further boost dose.
- For previously infected subjects:
  - T0 = baseline pre-vaccination after SARS-CoV-2 infection.
  - T1 = 8 days after the unique dose.
  - T2 = 1 month after the unique dose.
  - T3 = 3 months after the unique dose.
  - T6 = 6 months after the unique dose.
  - T9 = 9 months after the unique dose.
  - T boost = 8 days after the administration of a further boost dose.

Data regarding 150 healthcare workers of INT – IRCCS “Fondazione Pascale” Cancer Centre (27 and 123 with history/no history of COVID-19 infection, defined as infected and not infected subjects, respectively) were analyzed. Moreover, in order to exactly quantify and extend the analysis to a 9-month interval two subgroups were extrapolated for the previously COVID-infected (n = 3) and not infected subjects (n = 20). The study was performed under the statements of Declaration of Helsinki and approved by the local Institutional Ethics Committee. All participants gave their informed consent.

### Assay

Roche Elecsys Anti-SARS-CoV-2 S electrochemiluminescence immunoassay (ECLIA) for the in vitro quantitative determination of antibodies (including IgG) against spike RBD of SARS-CoV-2 in human serum was performed on Roche Cobas e 801 module. According to the manufacturer, the correlation test between Roche Elecsys Anti-SARS-CoV-2 S units per mL and WHO International Standards for anti-SARS-CoV-2 immunoglobulins showed an excellent correlation (*r*^2^ = 0.9992, slope = 0.972, intercept = 0.0072), thus allowing to consider specific Roche Elecsys Anti-SARS-CoV-2 S U/mL units equivalent to WHO International Standard BAU/mL (Binding Arbitrary Units per mL). Measuring range spanned from 0.4 BAU/mL to 250.0 BAU/mL, requiring a 1:10 dilution for samples with concentrations > 250.0 BAU/mL, extending the measuring range until to 2500.0 BAU/mL; values higher than 0.8 BAU/mL were considered positive. Serum samples with antibody titers > 2500.0 BAU/mL, from the selected subgroups above specified, were further diluted in order to obtain the exact quantitative concentration.

### Statistical analysis

Statistical analysis was performed by using the Statistical Package for Social Science (SPSS Inc., Chicago, IL, USA), version 27.0. Distribution of variables was evaluated by Shapiro–Wilk test; parametric data were represented as mean ± standard deviation (SD), whilst non-parametric variables were expressed as median (IR—Interquartile Range). Two-tailed Mann–Whitney for independent variables test was used to compare groups. Values lower than 0.4 BAU/mL were assumed as 0.4 and values higher than 2500.0 BAU/mL were reported as 2500.0; *p* values < 0.05 were considered statistically significant. Moreover, geometric mean titers (GMT) and relative fold changes (FC) were calculated. To overcome the novelty of general bead-based linear models, used to evaluate post-to-pre vaccination antibody titer increase or subsequent titer decline, the used statistic methods were extrapolated from Zaccaro et al. [17]. Different mathematical models were tested and evaluated by fitting median values extrapolated by immunoassays at different time-points for previously and not previously infected healthcare providers, in order to study and explain the antibody decline kinetics.

## Results

The enrolled cohort of healthcare workers (27 previously infected and 123 not infected) was represented as follows: 27 seropositive cases, 17 female and 10 male subjects with an overall mean age of 49.1 years (SD ± 8.9, range 34–65); and 123 seronegative cases, 80 female and 43 male subjects with an overall mean age of 46.2 years (SD ± 11.4, range 23–67) (Table 1).

**Table 1:** Demographic data of previously infected and not previously infected cohorts of healthcare providers

	Male (age)	Female (age)	Whole cohort (age)
	n (mean ± SD)	n (mean ± SD)	n (mean ± SD)
Previously infected subjects	10 (49.2 ± 8.9)	17 (49.3 ± 9.4)	27 (49.1 ± 8.9)
Not previously infected subjects	43 (46.4 ± 11.4)	80 (46.2 ± 11.3)	123 (46.2 ± 11.4)
Whole cohort	53 (46.9 ± 11.0)	97 (46.7 ± 11.1)	150 (46.7 ± 11.0)

Previously infected subjects = subjects previously infected with SARS-CoV-2, Not previously infected subjects = subjects not infected with SARS-CoV-2, n = number of subjects, mean = mean age, SD = standard deviation

**Fig. 1:**
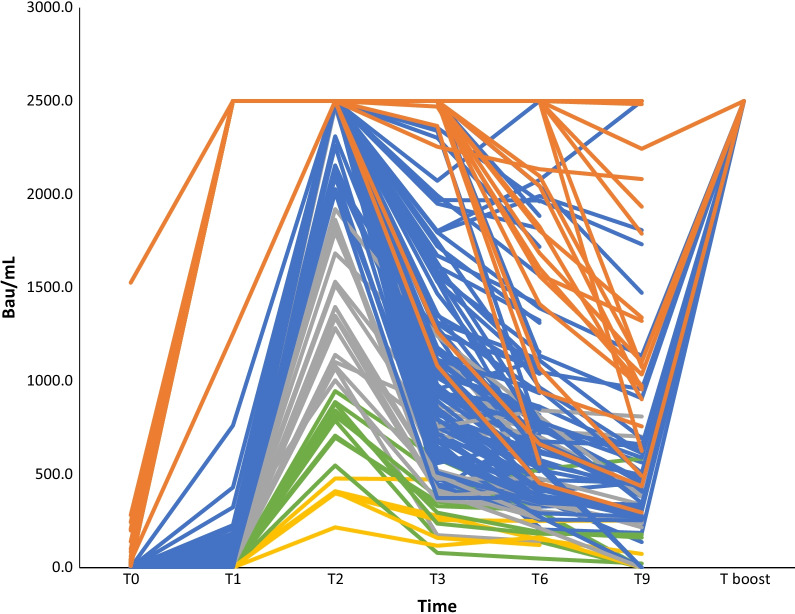
Vaccine immune response monitoring in previously infected and not previously infected cohorts of healthcare providers. For not infected subjects (n = 123): T0 = baseline pre-vaccination, T1 = 20 days after the first dose, T2 = 8 days after the second dose, T3 = 3 months after the second dose, T6 = 6 months after the second dose, T9 = 9 months after the second dose, T boost = 8 days after the administration of boost dose. For previously infected subjects (n = 27): T0 = baseline pre-vaccination after SARS-CoV-2 infection, T1 = 8 days after the unique dose, T2 = 1 month after the unique dose, T3 = 3 months after the unique dose, T6 = 6 months after the unique dose, T9 = 9 months after the unique dose, T boost = 8 days after the administration of a further boost dose. Orange lines: subjects previously infected with SARS-CoV-2. Yellow lines: subjects not previously infected and with T2 titers < 500.0 BAU/mL. Green lines: subjects not previously infected and with T2 titers < 1000.0 BAU/mL and ≥ 500.0 BAU/mL. Grey lines: subjects not previously infected and with T2 titers < 2000.0 BAU/mL and ≥ 1000.0 BAU/mL. Blue lines: subjects not previously infected and with T2 titers ≥ 2000.0 BAU/mL

Data regarding monitoring during the interval from baseline to boost dose administration are showed in Table 2 and Fig. 1. At each time-point, two-tailed Mann–Whitney U test showed significantly higher antibody titer concentrations in infected subjects in comparison with not previously infected ones, except for T boost at which previously infected workers presented higher (but not statistically significant) levels of antibodies.

**Table 2:** Vaccine immune response monitoring in previously infected and not previously infected cohorts of healthcare providers

	Median (IR) BAU/mL						
	T0	T1	T2	T3	T6	T9	T boost
Previously infected subjects	35.6	> 2500.0	> 2500.0	> 2500.0	2098.0	1212.5	> 2500.0
	(20.4–91.4)				(1565.5–>2500.0)	(957.2–2423.3)	
	n = 27	n = 27	n = 27	n = 27	n = 27	n = 26	n = 5
Not previously infected subjects	< 0.4	26.5	> 2500.0	853.4	574.0	381.0	> 2500.0
		(9.4–68.5)	(2020.0–>2500.0)	(514.9–1252.0)	(363.5–814.8)	(255.3–579.4)	
	n = 123	n = 123	n = 123	n = 123	n = 123	n = 62	n = 14

Previously infected subjects = subjects previously infected with SARS-CoV-2, Not previously infected subjects = subjects not infected with SARS-CoV-2, n = number of subjects, median = median of anti-SARS-CoV-2 S titers, IR = Interquartile Range of anti-SARS-CoV-2 S titers, For previously infected subjects: T0 = baseline pre-vaccination after SARS-CoV-2 infection, T1 = 8 days after the unique dose, T2 = 1 month after the unique dose, T3 = 3 months after the unique dose, T6 = 6 months after the unique dose, T9 = 9 months after the unique dose, T boost = 8 days after the administration of a further boost dose. For not infected subjects: T0 = baseline pre-vaccination, T1 = 20 days after the first dose, T2 = 8 days after the second dose, T3 = 3 months after the second dose, T6 = 6 months after the second dose, T9 = 9 months after the second dose, T boost = 8 days after the administration of boost dose

**Fig. 2:**
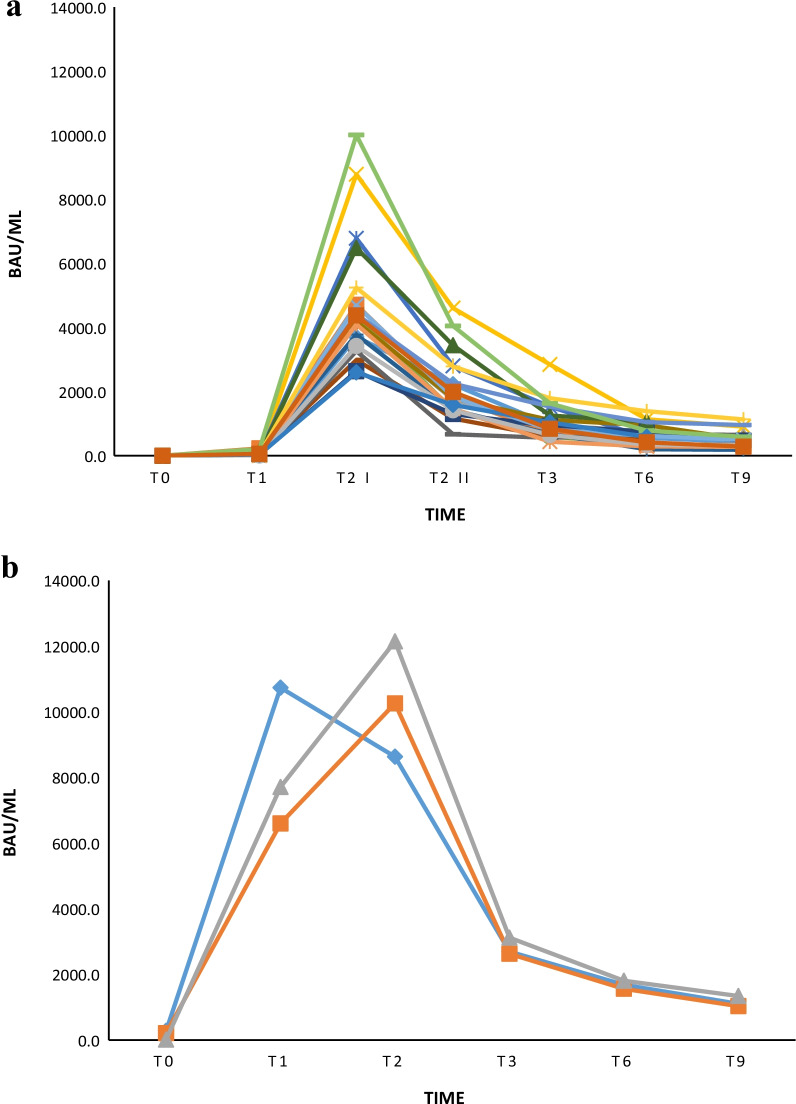
**a** Vaccine immune response decline in not previously infected healthcare providers’ diluted samples. T0 = baseline pre-vaccination, T1 = 20 days after the first dose, T2 I = 8 days after the second dose, T2 II = 1 month after the second dose, T3 = 3 months after the second dose, T6 = 6 months after the second dose, T9 = 9 months after the second dose. Each colored line represents a subject (n = 20). **b** Vaccine immune response decline in previously infected healthcare providers’ diluted samples. T0 = baseline pre-vaccination after SARS-CoV-2 infection, T1 = 8 days after the unique dose, T2 = 1 month after the unique dose, T3 = 3 months after the unique dose, T6 = 6 months after the unique dose, T9 = 9 months after the unique dose. Each colored line represents a subject (n = 3)

Despite the upper limit of 2500.0 BAU/mL imposed by the Roche method, a bimodal titer decline, before the further boost dose administration, was observed in most subjects. This finding was confirmed on data obtained from further diluted samples. In order to better define antibody titers’ trend in the interval immediately after the administration of the unique dose or the second dose, the T2 for previously infected subjects, and T2 I–T2 II time-points for not infected ones, respectively, were included (Table 3, Fig. 2a–b). Such analysis allowed to show for not previously infected workers a first rapid decline in the interval T2 I–T2 II with a negative slope of − 2495.5 followed by a progressive slow decline in the 6/9 month-period before the further vaccine boost (slope of − 847.3 in the interval T2 II–T3, − 342.2 in the interval T3–T6, − 123.1 in the interval T6–T9), with a slope of − 3342.9 in the interval T2 I–T3 and an overall negative slope of − 3808.2 from T2 I to T9 (Table 3, Fig. 2a). Peculiarly, in the cohort of previously infected workers, after an unexpected and unreported initial median increase of antibody titer in the T1–T2 interval (positive slope of 2550), a rapid decline followed by a slower decrease was observed (Table 3), with a negative slope of − 7562 in the interval T2–T3, − 1009 (T3–T6), − 582 (T6–T9) and an overall negative slope of − 9153 reported from T2 to T9 (Table 3, Fig. 2b). Both in previously infected cohort (n = 20) and in not previously infected one (n = 3), 2 different mathematical models were analyzed to study and calculate the antibody decline kinetics: an exponential *vs.* a power function model.

**Table 3:** Vaccine immune response in previously infected and not previously infected cohorts of healthcare providers’ diluted samples

	Median (IR) BAU/mL							
	T0	T1	T2 I	T2 II	T3	T6	T9	T boost
Previously infected subjects	207.4	7706.0	10,256.0		2694.0	1685.0	1103.0	52,694.5
	(108.5–244.2)	(7152.0–9220.5)	(9442.5–11,198.5)		(2661.0–2908.0)	(1623.5–> 1745.5)	(1069.0–1221.5)	(51,223.8–54,165.3)
	n = 3	n = 3	n = 3		n = 3	n = 3	n = 3	n = 2
Not previously infected subjects	< 0.4	52.3	4290.0	1794.5	947.2	605.0	481.8	33,419.5
		(30.7–81.0)	(3635.0–4844.3)	(1426.8–2381.0)	(671.8–1325.0)	(410.2–824.6)	(315.9–625.3)	(22,103.3–54,130.8)
	n = 20	n = 20	n = 20	n = 20	n = 20	n = 20	n = 16	n = 14

Previously infected subjects = subjects previously infected with SARS-CoV-2, Not previously infected subjects = subjects not infected with SARS-CoV-2, n = number of subjects, median = median of anti-SARS-CoV-2 S titers, IR = Interquartile Range of anti-SARS-CoV-2 S titers, For previously infected subjects: T0 = baseline pre-vaccination after SARS-CoV-2 infection, T1 = 8 days after the unique dose, T2 I = 1 month after the unique dose, T3 = 3 months after the unique dose, T6 = 6 months after the unique dose, T9 = 9 months after the unique dose, T boost = 8 days after the administration of a further boost dose. For not infected subjects: T0 = baseline pre-vaccination, T1 = 20 days after the first dose, T2 I = 8 days after the second dose, T2 II = 1 month after the second dose, T3 = 3 months after the second dose, T6 = 6 months after the second dose, T9 = 9 months after the second dose, T boost = 8 days after the administration of boost dose

**Fig. 3:**
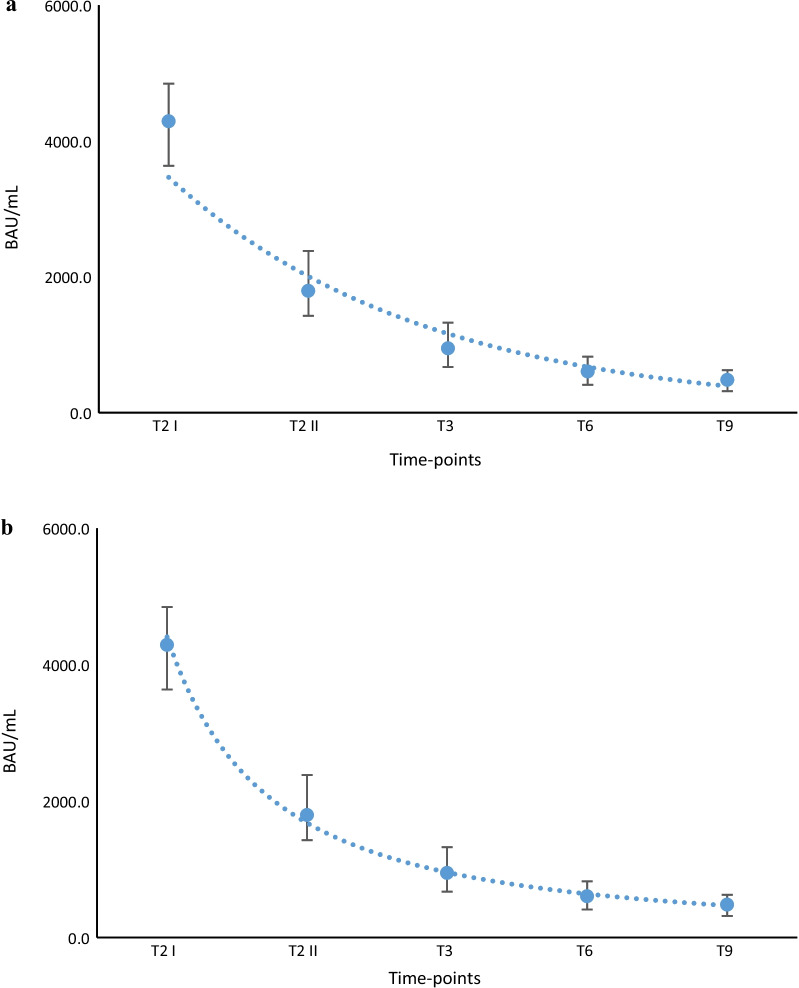
**a** Exponential model to study and calculate the antibody decline kinetics in not previously infected subjects. Blue dot line indicates the graphic representation of the exponential model, the single blue dots the median values at different time-points and black vertical lines the interquartile range of distributions. **b** Power function model to study and calculate the antibody decline kinetics in not previously infected subjects. Blue dot line indicates the graphic representation of the power function model, the single blue dots the median values at different time-points and black vertical lines the interquartile range of distributions

In not previously infected workers the following equations were detected:

$$\begin{array}{*{20}c} {{\text{y}} = 5982.8\,{\text{e}}^{{ - 0.546{\text{x}}}} ({\text{exponential}})} \\ {{\text{and}}} \\ {{\text{y}} = 4407.4\,{\text{x}}^{ - 1.392} ({\text{power}})} \\ \end{array}$$

for exponential and power function models, with *R*^2^ = 0.9502 and 0.9971, respectively (Fig. 3a–b).

**Fig. 4:**
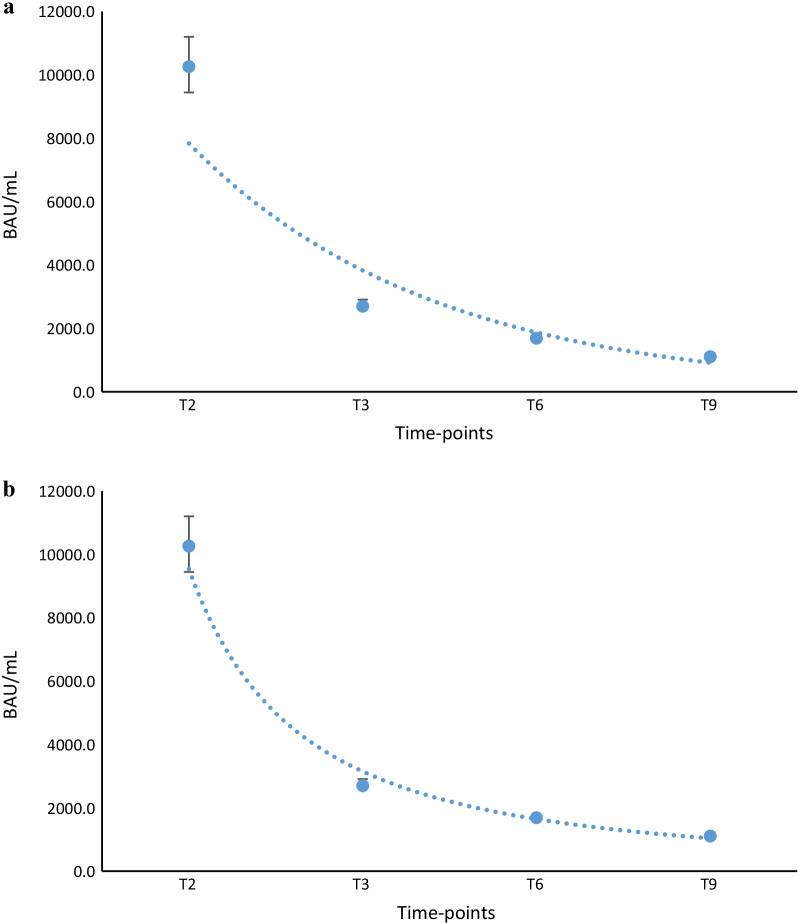
**a** Exponential model to study and calculate the antibody decline kinetics in previously infected subjects. Blue dot line indicates the graphic representation of the exponential model, the single blue dots the median values at different time-points and black vertical lines the interquartile range of distributions. **b** Power function model to study and calculate the antibody decline kinetics in previously infected subjects. Blue dot line indicates the graphic representation of the power function model, the single blue dots the median values at different time-points and black vertical lines the interquartile range of distributions

In previously infected subjects the following equations were detected:

$$\begin{array}{*{20}c} {{\text{y}} = 16028\,{\text{e}}^{{ - 0.716{\text{x}}}} ({\text{exponential}})} \\ {{\text{and}}} \\ {{\text{y}} = 9533.8\,{\text{x}}^{ - 1.599} ({\text{power}})} \\ \end{array}$$

for exponential and power function models, with *R*^2^ = 0.9136 and 0.988, respectively (Fig. 4a–b).

**Fig. 5:**
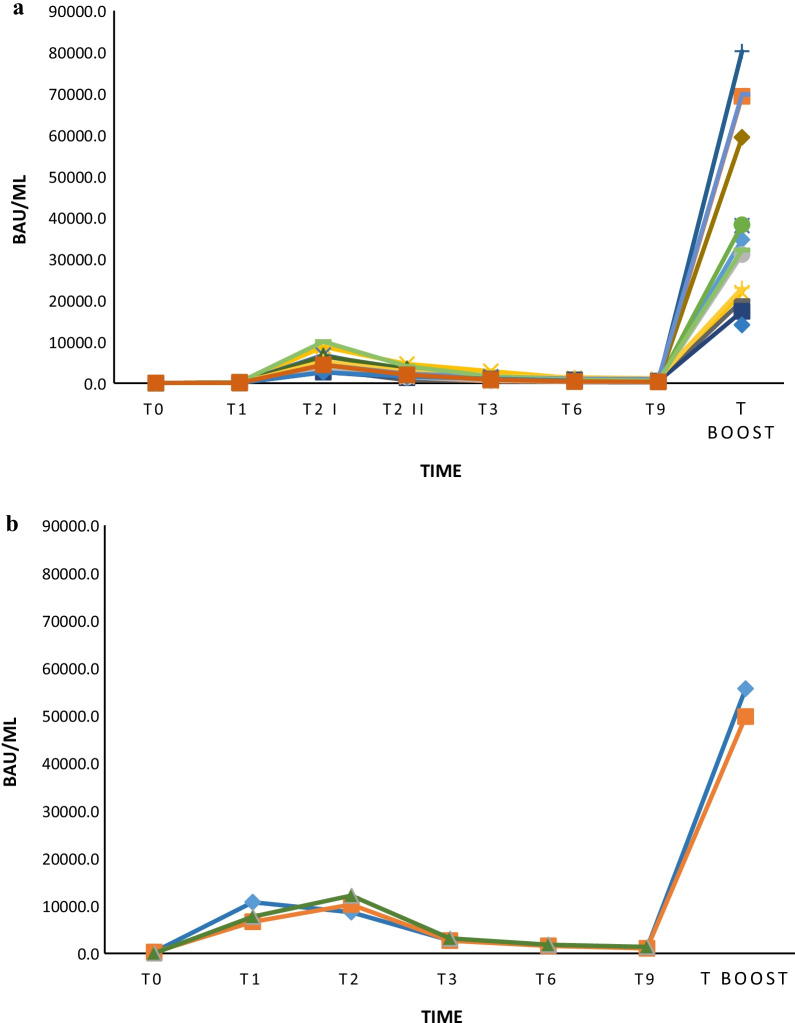
**a** Vaccine immune response increase after boost dose administration in not previously infected healthcare providers’ diluted samples. T0 = baseline pre-vaccination, T1 = 20 days after the first dose, T2 I = 8 days after the second dose, T2 II = 1 month after the second dose, T3 = 3 months after the second dose, T6 = 6 months after the second dose, T9 = 9 months after the second dose, T boost = 8 days after the administration of a further boost dose. Each colored line represents a subject. **b** Vaccine immune response increase after boost dose administration in previously infected healthcare providers’ diluted samples. T0 = baseline pre-vaccination after SARS-CoV-2 infection, T1 = 8 days after the unique dose, T2 = 1 month after the unique dose, T3 = 3 months after the unique dose, T6 = 6 months after the unique dose, T9 = 9 months after the unique dose, T boost = 8 days after the administration of a further boost dose. Each colored line represents a subject

By calculating GMT at each time-point and relative FC, it was found that the prolonged lower vaccine titer was about 1 log below in the 6/9-month interval after the single dose for previously infected individuals with SARS-CoV-2 and the two doses for those not previously infected (Table 4). The mean change in titer after the boost dose administration, on the other hand, was equal to or more than 1.5 FC higher than the T6–T9 time-points in both cohorts (1.5 and 1.7 higher than T6 and T9 for previously infected workers, and 1.7 and 1.9 higher than T6 and T9 for not previously infected ones, respectively) (Table 4). A mean increase of 0.7 and 0.9 FC was found comparing T boost–T2 time-points in infected subjects and in not previously infected ones, respectively (Table 4). At T boost time-point, previously infected workers presented higher (but not statistically significant) levels of antibodies in respect to not previously infected cohort (Table 4, Fig. 5a–b). At T boost time-point, however, a similar anti-S antibody titer was observed in previously infected and not-infected subjects suggesting that the vaccination boost was able to induce an equivalent immune response among the two groups eliminating any difference related to the previous infection.

**Table 4:** Antibody titers’ fold changes (FC) in previously infected and not previously infected cohorts of healthcare providers to monitor vaccine immune responses

Analyzed cohorts	Mean FC	Min FC	Max FC
*Previously infected subjects*			
T1 − T0	2.0	1.5	1.9
T2 − T1	0.1	− 0.1	0.2
T2 − T0	2.1	1.5	3.1
T3 − T2	− 0.6	− 0.6	− 0.5
T6 − T3	− 0.2	− 0.2	− 0.2
T9 − T6	− 0.2	− 0.2	− 0.1
T boost − T6	1.5	1.5	1.5
T boost − T9	1.7	1.7	1.7
T boost − T2	0.7	0.7	0.8
T boost − T0	2.3	2.3	2.4
*Not previously infected subjects*			
T1 − T0	1.6	0.6	2.4
T2 I − T1	2.0	1.3	3.0
T2 II − T2 I	− 0.4	− 0.7	− 0.2
T2 I − T0	− 0.3	− 0.5	− 0.1
T3 − T2 II	− 0.2	− 0.5	0.1
T6 − T3	− 0.1	− 0.3	0.0
T9 − T6	− 0.1	− 0.3	0.0
T boost − T6	1.7	1.2	2.6
T boost − T9	1.9	1.3	2.6
T boost − T2 I	0.9	0.4	1.3
T boost − T2 II	1.2	0.7	1.8
T boost − T0	4.5	4.1	4.9

Previously infected subjects = subjects previously infected with SARS-CoV-2, Not previously infected subjects = subjects not infected with SARS-CoV-2, For previously infected subjects: T0 = baseline pre-vaccination after SARS-CoV-2 infection, T1 = 8 days after the unique dose, T2 I = 1 month after the unique dose, T3 = 3 months after the unique dose, T6 = 6 months after the unique dose, T9 = 9 months after the unique dose, T boost = 8 days after the administration of a further boost dose. For not infected subjects: T0 = baseline pre-vaccination, T1 = 20 days after the first dose, T2 I = 8 days after the second dose, T2 II = 1 month after the second dose, T3 = 3 months after the second dose, T6 = 6 months after the second dose, T9 = 9 months after the second dose, T boost = 8 days after the administration of boost dose, mean FC = mean fold changes of anti-SARS-CoV-2 S mean geometric titers, min FC = minimum value of fold changes of anti-SARS-CoV-2 S mean geometric titers, max FC = maximum value of fold changes of anti-SARS-CoV-2 S mean geometric titers, n = number of subjects

## Discussion

In accordance with first data reported in literature [11, 13, 18], our findings confirm that administration of a single dose to previously infected subjects is sufficient to elicit an adequate immunoresponse, the latter resulting more sustained in terms of concentration and duration if compared to antibody response of not previously infected cohort. Nevertheless, for the first time in literature, we demonstrated a bimodal decline kinetic characterized by a very rapid first decline from the highest antibody peak (1 month after the unique vaccine dose in previously infected workers and 8 days after the second dose in not previously infected one), followed by a slow decline which ensures a long-term immune protection lasting more than 6 months. Our data fit a power function decline curve, which allows the calculation and identification of a specific trend that can predict the decline rate of anti-RBD antibody titer, surrogate of neutralizing protective antibodies [19, 20], with excellent accuracy in both previously infected and uninfected individuals. Moreover, the initial more pronounced decline observed in previously infected subjects (demonstrated by higher negative slopes) was counterbalanced by the less robust response to the additional boost dose, resulting in overlapping of antibody titers in the 2 different cohorts 8 days after boost vaccine administration (T boost).

The apparent absence of negative effects related to the rapid decline on the vaccine's immune protective role has been referred to a large majority of low avidity antibodies induced in the early post-vaccination stages [21–23]. High avidity antibodies with prolonged anti-transmission efficacy show a longer half-life and are lost over a longer interval period. Protective humoral immune response is generally characterized by a parabolic trend with a protective plateau, several months long, following natural infection as well as vaccine induced active immunity, also for the latest developed subunits vaccines. As paradigm the long-lasting immune response following a single dose of L1-based Virus-like particles (VLPs), the anti- DNA papillomavirus (HPV) vaccine [24], and following multiple doses of a recombinant E2-based vaccine against the RNA-hepatitis E virus (HEV) [25] can be considered. The trend observed in the current anti-SARS-CoV-2 vaccination shows, instead, after a rapid increment an asymptotic hyperbolic trend towards a modest not-protective immune response, with higher risk of further infections, gradually declining below a titer which should represent the protective immune threshold. The high titer stage is lost in less than 30 days and is referred mainly to low-avidity antibodies, leaving the protective activity to the low-titer stage ranging from 1893.4 to 469.5 BAU/mL for the subject without previous COVID infection and from 2806.4 to 1152.2 BAU/mL for those with a previous COVID infection (GMT from Fig. 2a and b, respectively). The alternative dramatic inference would be that the protective period is just a month.

The identification and quantification of the protective lower antibody level, whose avidity along with neutralizing activity increases over time [26], becomes relevant for monitoring the individual protection level and selecting the appropriate further boosting time. Moreover, it becomes a reference value to compare the efficacy of other vaccines as well as their delivery and formulation (including adjuvants) besides the possibility to develop pre-vaccination strategies to improve the immune response to vaccines [27]. The limitation of the current study is the low number (n = 3) of previously infected vaccinees. Such number limit, however, is not crucial for this specific manuscript given that the immunoresponse decline of the previously infected follows the same curve (although starting from a higher antibody titer) of the non-previously infected vaccinees. It suggests that also the humoral immune response following the vaccination is very similar for the lower antibody level with a FC of 0.4 between the previously infected and non-previously infected at 9 months after the vaccination.

Fortunately, the cellular immunity, capable of preventing severe clinical diseases, seems to last much longer. The complementary immune response (short-lived humoral immunity *vs* long-lasting cellular immunity), although effective in limiting overall mortality, with the consequent reduction of pressure in the ICU, does not prevent and does not protect against transmission of SARS-CoV-2, resulting in high circulation of the virus among unvaccinated subjects, including the younger population, and the continuous production of variants characterized by changes in transmissibility and pathogenicity. The high mutation rate, peculiar to the RNA virus, can however lead to a dual opposite result: selection of defective and less efficient viruses up to extinction [28, 29]; risk of more efficiently transmitted variants as the current omicron pandemic [30, 31].

## Conclusions

In conclusion, further extended analyses are mandatory to confirm our preliminary findings in larger cohorts of subjects (including patients and immunocompromised individuals), in order to monitor antibody titers also after the administration of further boost doses, verify the protective borderline levels of immunity and define the optimal administration schedule of vaccine boosters. Our results, highlighting the current bimodal antibody-titer decline after BNT162b2 mRNA anti-SARS-CoV-2 vaccination, can contribute to such goal, besides a direct comparison of other FDA-approved and candidate vaccines.

## Data Availability

All relevant data and their evaluation are reported in the manuscript.

## Abbreviations

ACE2: Human Angiotensin Converting Enzyme 2
AIFA: Agenzia Italiana del Farmaco, the Italian regulatory agency for drugs
BAU/mL: Binding Arbitrary Units per mL
COVID-19: Coronavirus disease 2019
ECLIA: Electrochemiluminescence Immunoassay
FC: Fold Change
GMT: Geometric Mean Titer
HPV: Human papilloma virus
HEV: Hepatitis E Virus
ICU: Intensive Care Units
IR: Interquartile Range
RBD: Receptor-Binding Domain
S: Trimeric spike glycoprotein
SARS-CoV-2: Severe Acute Respiratory Syndrome caused by Coronavirus 2
SD: Standard Deviation
Tx: Time Points

## For not infected subjects

T0: Baseline pre-vaccination
T1: 20 Days after the first dose
T2: 8 Days after the second dose, distinguishing
T2 I: 8 Days after the second dose
T2 II: 1 Month after the second dose
T3: 3 Months after the second dose
T6: 6 Months after the second dose
T9: 9 Months after the second dose
T boost: 8 Days after the administration of a further boost dose

## For previously infected subjects

T0: Baseline pre-vaccination after SARS-CoV-2 infection
T1: 8 Days after the unique dose
T2: 1 Month after the unique dose
T3: 3 Months after the unique dose
T6: 6 Months after the unique dose
T9: 9 Months after the unique dose
T boost: 8 Days after the administration of a further boost dose
VLP: Virus-like particles
